# The inflammatory injury in the striatal microglia-dopaminergic-neuron crosstalk involved in Tourette syndrome development

**DOI:** 10.3389/fimmu.2023.1178113

**Published:** 2023-04-28

**Authors:** Xueming Wang, Xiumei Liu, Liangliang Chen, Xiaoling Zhang

**Affiliations:** ^1^ Plastic Surgery Department, Fujian Children’s Hospital, Fuzhou, China; ^2^ Fujian Maternity and Child Health Hospital, Affiliated Hospital of Fujian Medical University, Fuzhou, China; ^3^ Developmental and Behavior Pediatrics Department, Fujian Children’s Hospital, Fuzhou, China; ^4^ Child Healthcare Department, Fuzhou Maternal and Child Health Hospital, Fuzhou, China

**Keywords:** Tourette syndrome, microglia, M1 polarization, striatum, dopaminergic neuron

## Abstract

**Background:**

Tourette syndrome (TS) is associated with immunological dysfunction. The DA system is closely related to TS development, or behavioral stereotypes. Previous evidence suggested that hyper-M1-polarized microglia may exist in the brains of TS individuals. However, the role of microglia in TS and their interaction with dopaminergic neurons is unclear. In this study, we applied iminodipropionitrile (IDPN) to establish a TS model and focused on the inflammatory injury in the striatal microglia-dopaminergic-neuron crosstalk.

**Methods:**

Male Sprague–Dawley rats were intraperitoneally injected with IDPN for seven consecutive days. Stereotypic behavior was observed to verify the TS model. Striatal microglia activation was evaluated based on different markers and expressions of inflammatory factors. The striatal dopaminergic neurons were purified and co-cultured with different microglia groups, and dopamine-associated markers were assessed.

**Results:**

First, there was pathological damage to striatal dopaminergic neurons in TS rats, as indicated by decreased expression of TH, DAT, and PITX3. Next, the TS group showed a trend of increased Iba-1 positive cells and elevated levels of inflammatory factors TNF-α and IL-6, as well as an enhanced M1-polarization marker (iNOS) and an attenuated M2-polarization marker (Arg-1). Finally, in the co-culture experiment, IL-4-treated microglia could upregulate the expression of TH, DAT, and PITX3 in striatal dopaminergic neurons *vs* LPS-treated microglia. Similarly, the TS group (microglia from TS rats) caused a decreased expression of TH, DAT, and PITX3 compared with the Sham group (microglia from control rats) in the dopaminergic neurons.

**Conclusion:**

In the striatum of TS rats, microglia activation is M1 hyperpolarized, which transmits inflammatory injury to striatal dopaminergic neurons and disrupts normal dopamine signaling.

## 1 Introduction

Tourette syndrome (TS) is a childhood-onset developmental neurological disease characterized by motor and vocal behavioral stereotypes (1). The estimated pooled prevalence rate of TS is 0.53%, with a male predominance (2–4). About 1% of school-age children are affected by TS, and boys are approximately four times more likely to develop TS than girls (5, 6). The main affected brain regions include the basal ganglia and the related corticostriatal-thalamocortical (CSTC) circuit (7) or the substantia nigra-striatum network. In this circuit, it is widely accepted that dopamine (DA) is the main excitatory neurotransmitter, and the DA system, including the important factor dopamine transporter (DAT), can affect locomotion behavior, and theoretically, it plays an important role in the pathophysiology of TS (8). Besides DA, TS associated abnormalities in neurotransmission include glutamate (Glu) and gamma-aminobutyric acid (GABA) (9, 10).

Microglia-mediated immune overactivation is an important cause of central nervous system injury. Normally, under the condition of microglia, they can polarize into either the M1 (pro-inflammatory type) or M2 (anti-inflammatory type) phenotype in response to different micro-environmental disturbances (11). M1 polarization has a variety of biological functions but often damages adjacent neurons through inflammatory cytokines and the corresponding neurotoxicity (12–15). Previous evidence has suggested that hyper-M1-polarized microglia may exist in the brains of TS individuals (16–19). However, there is a lack of direct evidence to reveal the role of microglia in TS, as well as its downstream interaction with dopaminergic neurons (considering the DA system is closely related to TS development or behavioral stereotypes) (20–25).

Iminodipropionitrile (IDPN) is a synthetic organic nitrile, and it is the most commonly used inducer of TS, which has a long-term effect (19). We have used this model to observe changes in different systems and tissues (26). Peripherally, IDPN triggers immune dysfunction through impairment of mature Th cells, especially the Treg subset. In this study, we probed the role of striatal microglia-dopaminergic-neuron crosstalk in a rat model of TS induced by IDPN. For the first time, we confirmed that neuroinflammation triggered by M1 polarization of microglia can cause significant injuries to dopaminergic neurons in the striatum of TS rats.

## 2 Materials and methods

### 2.1 TS model

Male Sprague–Dawley rats (6–8 weeks, weight 200 g) were used for TS model establishment. The TS group was intraperitoneally injected with iminodipropionitrile (IDPN, 300 mg/kg/day, Sigma-Aldrich, USA) for seven consecutive days, and the control group was intraperitoneally injected with saline (5 ml/kg/day) for 7 days. The TS model was verified by stereotypical behaviors. If the rats had a stereotypic score lower than 1, the sample was removed.

After behavioral observation, each rat was sacrificed, and the striatal tissue was isolated. A part of the striatal tissues was fixed in 4% paraformaldehyde, the remaining tissues were homogenized, and protein was extracted for ELISA and Western blot assays. Another batch of animals was used for the purification of microglia, and these microglia were named the Sham group or TS group in the cell experiment.

The above animal experiments were approved by the ethics committee of Fujian Maternity and Child Health Hospital.

### 2.2 Co-culture of striatal dopaminergic neurons and microglia

First, a primary culture of dopaminergic neurons was performed. The ventral midbrain region containing the striatum was dissected and washed three times with Hank’s balanced salt solution containing 10 mM HEPES and 20 mM glucose. The tissue was mechanically dissociated at room temperature and suspended in Neurobasal medium (Gibco, Invitrogen, Carlsbad, CA, USA) containing 2 mM glutamine and B27 supplement (Gibco, Invitrogen). Cells were seeded on poly-D-lysine/laminin-coated plates (10^5^ cells/well) and cultured in Neurobasal medium (Gibco, Invitrogen, Carlsbad, CA, USA) containing 2 mM glutamine and 2% B27 supplement (Gibco, Invitrogen) at 37°C in a humidified 5% CO_2_ atmosphere. Half of the medium was changed every 2 days until treatment. Co-culture was performed after 7 days of seeding. For each well, an equal amount of microglia (10^5^ cells) from different groups was added; after 48 h, the microglia were washed out slightly, and the adherent dopaminergic neurons were collected for the assay. The added microglia were divided into the following groups: For different types of stimulation, the LPS group (of microglia) was added LPS (1 μg/ml), and the IL-4 group was added IL-4 (20 μg/ml), and a 24-hour treatment was allowed before microglia collection. Next, two groups of microglia were added to the pool of dopaminergic neurons. In the other batch, an equal amount of microglia from the TS or control rats (namely, the TS group or Sham group) was added.

### 2.3 Immunohistochemical and immunofluorescence staining

For IHC staining, the tissues were embedded in paraffin and cut into 5 mm sections. Sections were mounted on slides. The sections of striatum were subjected to antigen, followed by antibody hybridization (the primary antibody was the rabbit anti-Iba-1 antibody, and the secondary antibody was the mouse anti-rabbit antibody). Targets were visualized by 3,3-diaminobenzidine. For immunofluorescence staining, tissues were fixed in 4% paraformaldehyde overnight, dehydrated in 20% sucrose (0.1 M PBS) for 24 h at 4°C, and further dehydrated in 30% sucrose (0.1 M PBS) for 24 h at 4°C. The sections were cut into 15-μm sections on a cryostat. The sections were rinsed in 0.01 M PBS and blocked for 2 h with donkey serum (in 0.3% Tween-20 and 0.01 M PBS) and then incubated with the primary antibodies at 4°C overnight (1:500). Subsequently, sections were washed three times in 0.01 M PBS for 5 min and incubated with several conjugates with FITC (1:200) or CY3 (1:200). Next, sections were incubated with DAPI for nucleus staining for 15 min and washed three times for 5 min each. Finally, sections were cover-slipped, and images were captured under a fluorescence microscope. For cellular immunofluorescence, cells were fixed with 4% PFA for 30 min, washed with 0.01 M PBS, permeabilized with 0.1% Triton X-100 for 5 min, and blocked with goat serum for 1 h. Labeling was performed by incubating cells for 1 h with specific rabbit antibodies (including CD86, Arg-1, TH, DAT, and PITX3). After three washes (using PBS), cells were incubated with the specific secondary antibody (Alexa594-conjugated goat anti-rabbit antibody, Life Technologies). Cells were washed three times with PBS and incubated in DAPI for 5 min. Samples were observed under a fluorescence microscope.

### 2.4 ELISA assay

Rat striatal tissues were homogenized in a lysis buffer with protein inhibitors and PMSF (1 mM). The lysates were centrifuged at 1,000 rpm for 5 min, and the supernatant was stored at 2 to 8°C. The above samples were used to detect the concentration of inflammatory factors (TNF-α, IL-6, and IL-10), and microglia polarization markers (iNOS or Arg-1), by ELISA. All ELISA operations followed the official instructions. Briefly, 50 μl of the sample was added to each well, and the standard curve was plotted based on the corresponding OD values. The concentration of each inflammatory factor was calculated according to the curve equation.

### 2.5 Western blotting

The tissues or cells were lysed using an ice-cold lysis buffer (50 mM Tris, pH 7.4, 150 mM NaCl, 1% SDS, 1 mM EDTA, 1% NP-40) containing 1 mM protein inhibitor and 1 mM PMSF. The lysates were centrifuged at 10,000×*g* at 4°C for 10 min, and the supernatants were collected. Protein concentration was measured using the BCA protein assay. Equal amounts of protein were separated using 10% SDS-PAGE before being transferred to PVDF membranes. Membranes were incubated with primary mouse antibodies or anti-GAPDH antibodies (Santa Cruz Biotechnology, Santa Cruz, CA, USA). The blotting was developed using HRP-conjugated goat anti-mouse IgG (Santa Cruz Biotechnology) and detected by an ECL kit (Amersham, Piscataway, NJ, USA). The applied primary antibodies were as follows: rabbit anti-TH (1:500), rabbit anti-DAT (1:500), rabbit anti-PITX3 (1:250), rabbit anti-iNOS (1:500), and rabbit anti β-actin (1:10,000).

### 2.6 Statistical analysis

Results were expressed as means ± standard error. For comparison between two groups, Student’s t-test was used after the normal distribution test; for groups with unequal variance, Welch’s t-test was used for comparison. For more than two groups, one-way ANOVA was used; nonparametric tests were used when data were not normally distributed. Additionally, Bonferroni correction was used to control family-wise error rates. A *P* value <0.05 was considered statistically significant.

## 3 Results

### 3.1 Pathological damage to striatal dopaminergic neurons in TS rats

**Figure 1:**
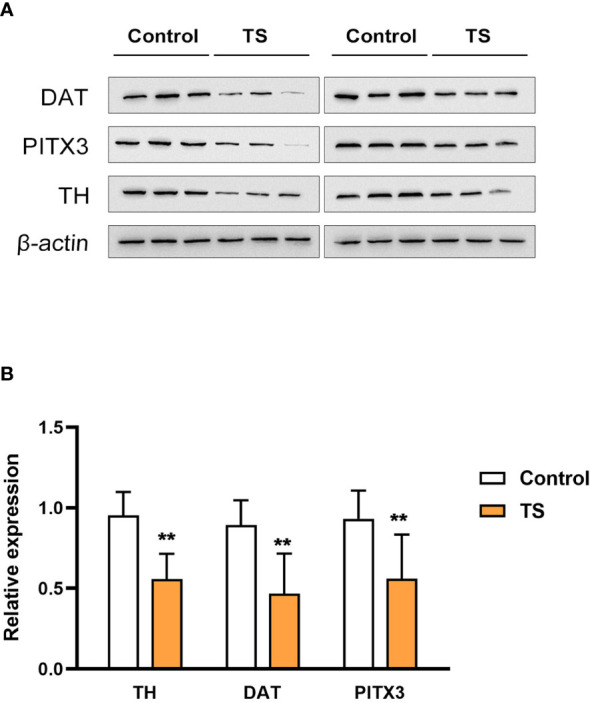
The expression of tyrosine hydroxylase (TH), dopamine transporter (DAT), and PITX3 is significantly decreased in the striatum of the Tourette syndrome (TS) rats. **(A)** The blots of TH, DAT, and PITX3. **(B)** The statistical analysis of the protein expression. **P <0.01.

First, the TS model was validated with respect to stereotypical behavior induced by IDPN. The IDPN-treated rats had significantly more counts of biting, head twitching, shaking claws, and continuous rotation, and the stereotypic behavior score was significantly increased. The detailed behavioral changes have been reported in our previous work (27). To assess the damage to striatal dopaminergic neurons, three associated markers were used: tyrosine hydroxylase (TH, the classic marker of striatal dopaminergic neurons), dopamine transporter (DAT, an important marker in the presynaptic membrane in dopaminergic neurons), and PITX3 [involved in the production, maintenance, and survival of dopaminergic neurons, as well as an important and specific transcription factor in the development of midbrain dopaminergic neurons (28)]. We found that the expression of TH, DAT, and PITX3 were significantly decreased in the striatum of TS rats (assessed by Western blotting, **Figures 1A, B**), which suggests that IDPN injection can induce pathological damage to striatal dopaminergic neurons.

### 3.2 M1 over-polarization of striatal microglia is involved in TS

**Figure 2:**
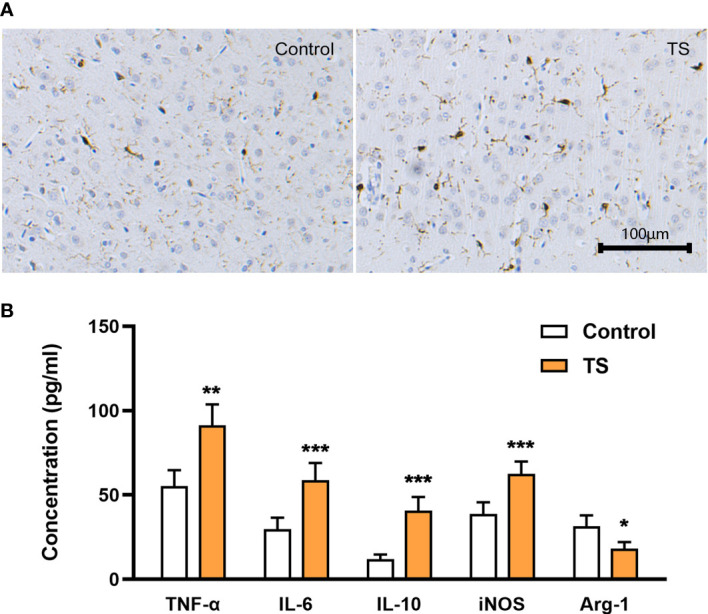
Activation of striatal microglia is involved in TS. **(A)** The TS group has a trend toward increased Iba-1 positive cells, with asymmetrical branches and an amoeba-like appearance. **(B)** The levels of inflammatory factors TNF-α, IL-6, IL-10, and iNOS are significantly increased in the striatum of TS individuals, while the M2 marker Arg-1 is decreased. *P <0.05, **P <0.01, ***P <0.001.

A possible mechanism of TS-related injuries in striatal dopaminergic neurons may be the activation (especially polarization) of striatal microglia. First, the TS group showed a trend of increased Iba-1 positive cells with asymmetrical branches and an amoeba-like appearance (**Figure 2A**). Meanwhile, ELISA assays showed that the levels of inflammatory factors TNF-α and IL-6 were significantly increased (P <0.01, **Figure 2B**), and intriguingly, the anti-inflammatory factor IL-10 level was also increased (P <0.01, **Figure 2B**) in the striatum of TS individuals. In addition, iNOS (the M1-polarization marker) expression was elevated (*P <*0.01; **Figure 2B**) and Arg-1 (the M2-polarization marker) expression was decreased (*P <*0.01; **Figure 2B**) in the striatum. Taken together, there is a M1 over-polarization of microglia in the striatum of TS individuals, and the associated inflammatory signaling may be involved in the central mechanism of TS development.

### 3.3 The possible microglia-neuron crosstalk in TS

**Figure 3:**
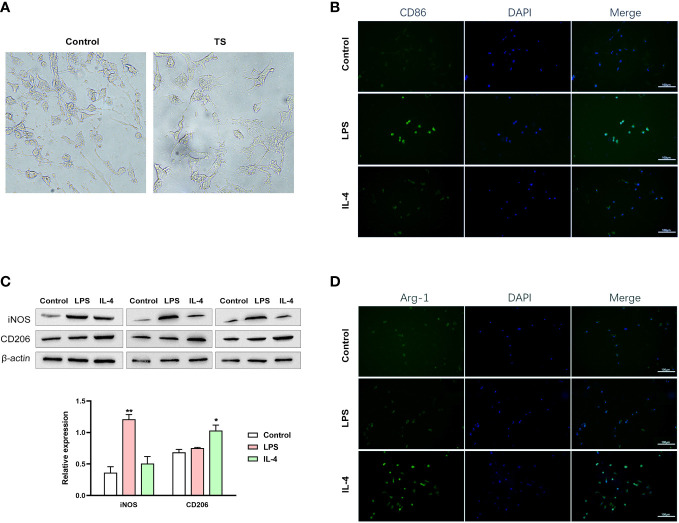
Different groups of microglia. **(A)** The morphology of microglia in two groups. **(B–D)** Normal microglia are treated using lipopolysaccharide (LPS) (towards the M1 phenotype) or IL-4 (towards the M2 phenotype). **(B)** LPS treatment effectively induced a M1 polarization of microglia (verified by the IF of the marker CD86). **(C)** IL-4 triggered a M2 polarization (verified by the IF of the marker Arg-1). **(D)** Western blot analysis of iNOS and CD206 expression in two treatment groups. *P <0.05, **P <0.01.

**Figure 4:**
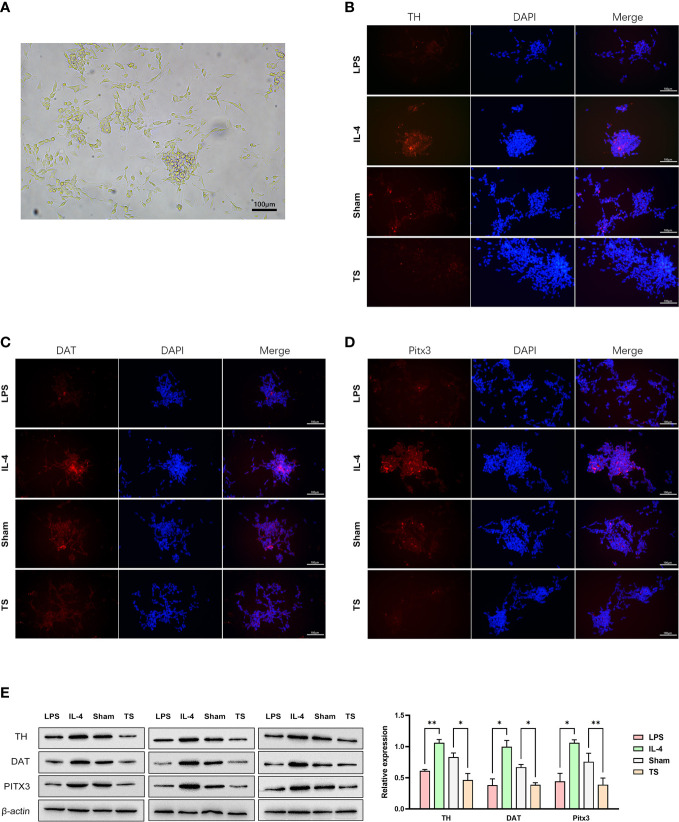
Striatal dopaminergic neurons were co-cultured with microglia of different polarization statuses. **(A)** Striatal dopaminergic neurons are cultured. **(B)** Immunofluorescence staining of TH expression in different groups: co-cultured with LPS-treated microglia, IL-4 treated microglia, control-animal-derived striatal microglia, and TS-rat-derived microglia. **(C)** Immunofluorescence staining of DAT expression in different groups. **(D)** Immunofluorescence staining of PITX3 expression in different groups. **(E)** Western blot analysis of the expression of TH, DAT, and PITX3 in different co-culture groups. *P <0.05, **P <0.01.

Based on the M1 over-polarization of microglia, it is reasonable to assume that the inflammatory crosstalk between microglia and neurons may be an important pathogenic mechanism for TS-related injuries. Therefore, we isolated microglia from two groups of animals. After 4 days of purification culture, the morphology of microglia (**Figure 3A**) in two groups was as follows: most of the cells in Sham group had small, narrow, and long cytosol, few amoeboid like cells; while the morphology of microglia in TS group was diverse, many cells had asymmetric branches, and most of them were amoeboid like cells. Next, we stimulated normal microglia using LPS (towards M1 phenotype) and IL-4 (towards M2 phenotype), and the striatal dopaminergic neurons were co-cultured with different groups of microglia. As expected, LPS treatment effectively induced a M1 polarization of microglia (verified by the marker CD86 and increased iNOS expression, **Figures 3B, D**), and IL-4 triggered a M2 polarization (verified by the marker Arg-1 and increased CD206 expression, **Figures 3C, D**). Subsequently, striatal dopaminergic neurons were cultured *in vitro* (**Figure 4A**), and they were co-cultured with microglia of different polarization status for 48 h. The expression of TH, DAT, and PITX3 was determined after co-culture. The IL-4 treated microglia induced higher levels of TH, DAT, and PITX3 in neurons, as observed by immunofluorescence staining (**Figures 4B-D**) and Western blotting (**Figure 4E**). Similarly, striatal dopaminergic neurons were co-cultured with microglia from different rats (the Sham group or TS group), and the TS-group microglia (*vs* the Sham-group) induced a decreased expression of TH, DAT, and PITX3 in the dopaminergic neurons (**Figures 4B-E**). These findings, at least partially, indicate that the decreased TH, DAT, and PITX3 may be caused by the crosstalk with the M1-type microglia in the TS individual.

## 4 Discussion

**Figure 5:**
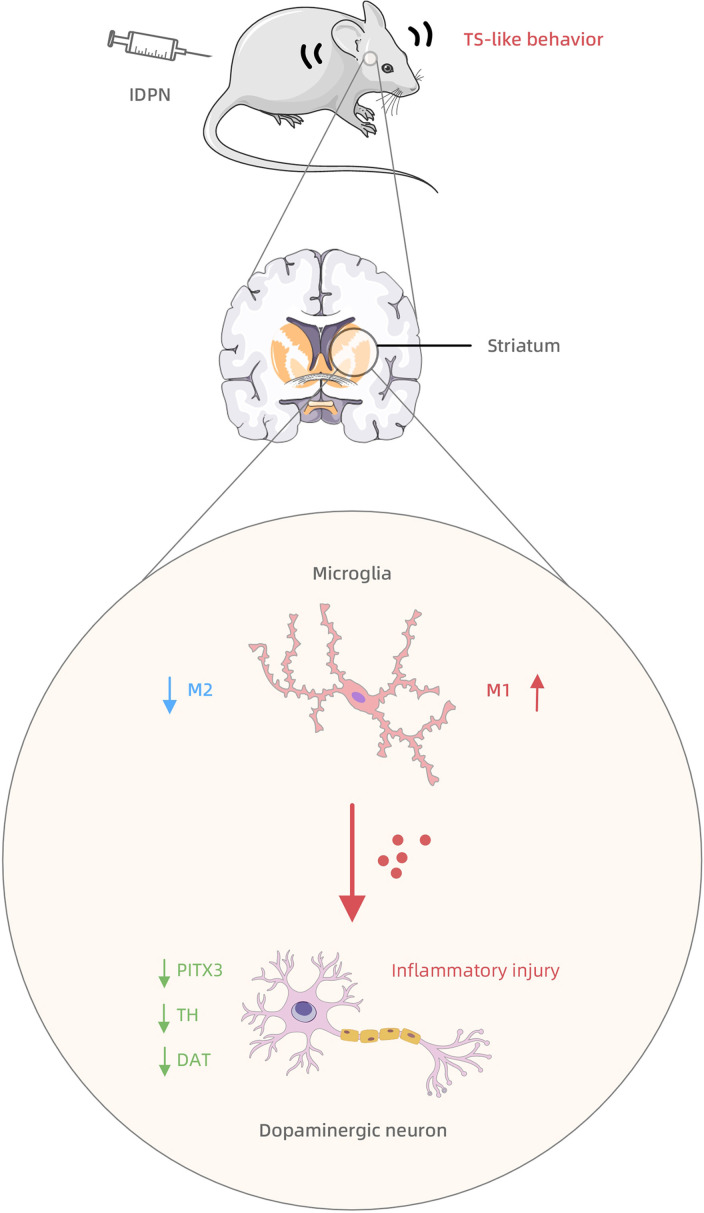
The mechanism summary diagram of IDPN-induced TS development involving the striatal microglia-dopaminergic-neuron crosstalk.

In this study, we observed inflammatory injury to striatal dopaminergic neurons in TS rats. We also observed an increased M1 polarization and decreased M2 polarization of the striatal microglia and the interaction between microglia and dopaminergic neurons, which may transmit the inflammatory injury. Based on these findings, we tentatively propose a hypothesis for the pathogenesis of TS (as shown in **Figure 5**): in the striatum of TS rats, microglia are hyperactivated, with excessive M1 polarization and overexpression of inflammatory factors that cause sustained neurotoxicity to striatal dopaminergic neurons, which drive the development of stereotypical behaviors.

Neuroinflammation plays a crucial role in the pathophysiology of neural disorders. Microglia activation is a major event following central nervous system inflammation. In particular, M1 polarization has been regarded as the biological basis for many abnormal behaviors and neuropsychiatric disorders, especially those associated with neuroinflammation (29–33). The imbalanced M1/M2 polarization can cause neurological disorders in all possibilities (34, 35), and many scholars were attempting to answer why microglia kill neurons after neural disorders (36). Increasing studies have reported that the neuroinflammation caused by microglia in the striatum mediates various central diseases, such as Parkinson’s disease (37), schizophrenia (38), Alzheimer’s disease (39, 40), and other negative affective conditions (41); and the potential molecular mechanisms include IL-13 (42), IL-6 (41), TREM2 (39), BIN1 (39), and toll-like receptors (36). As far as we know, only a few studies have shown that the neuroimmune response mediated by microglia may be involved in TS (16–19) and the associated molecular mechanism include L-6, TNF-α, CD45, IFN-γ, histamine, etc. (43–49). We here highlight the role of microglia polarization in TS development and confirm the increased M1 type and decreased M2 type, which have not been fully reported previously [instead, known studies have mainly focused on T cells (50)].

Further, we here innovatively discover that striatal dopaminergic neurons are the key cell targets of microglia in TS. This is in line with previous conclusions. The striatal dopaminergic neurons are vulnerable to neurotoxicity (including different products of microglia) (51, 52), and their functions are involved in Parkinson’s disease and Huntington’s chorea (53, 54). Robust dopaminergic neuron function helps maintain a healthy microenvironment in the brain. For example, the cerebral dopamine neurotrophic factor possesses immune-modulatory properties that benefit brain diseases (37). Furthermore, many studies have indicated that stereotypic behavior is associated with the dopaminergic system (55–58). Moreover, the striatum is indeed one of the most important brain regions associated with the pathogenesis of TS (59). DAT plays a role in many neurodevelopmental diseases, e.g., altered DA availability mediated through DAT may affect autistic traits in autism spectrum disorders (60–62). The loss of DAT affects the reuptake of DA and causes an accumulation of DA in the synaptic cleft, which triggers an exorbitant DA signal and drives pathological stereotypic behavior. The current study further discovers that decreased DAT, as well as TH and PITX3, may be the result of inflammatory injuries delivered by microglia. The deficiency in tyrosine hydroxylase (TH) function is closely linked to neurodevelopmental behavioral disorders (63). The decreased TH expression (STX1A+/TH+ cells) in the striatum of TS individuals has also been observed in our previous article (27), which is a clear indicator of dopaminergic neuron injury. PITX3 is a homeodomain-containing transcription factor belonging to the pituitary homeodomain family. It is involved in the production, maintenance, and survival of dopaminergic neurons. The PITX3 deficiency can lead to the loss of the substantia nigra striatum path, the deprivation of dopaminergic neurons in the substantia nigra, and the impairment of dopaminergic development, which may drive the development of Parkinson’s disease (64). In a TH-Cre/Pitx3-fl/fl (Pitx3cKO) mouse model, it was noticed that Pitx3 deficiency promotes age-dependent alterations in striatal medium spiny neurons (65). Although many studies have shown the link between PITX3 and Parkinson’s disease, the clear association with TS is largely unknown. This work is the first to show a decreased striatal PITX3 expression in TS rats, which suggests a potential role for PITX3 in the pathogenesis of TS.

Still, the present study has some limitations. First, some of the findings of this study are inconsistent with previous studies, and the exact reasons for these inconsistencies are unclear. For example, we noticed that the IL-4 treatment is beneficial for dopaminergic neurons in comparison with LPS. IL-4, a well-known anti-inflammatory cytokine, is expressed in microglia in the brain. It can regulate the polarization of the peripheral macrophage phenotype and inhibit the production of inflammatory mediators, such as interleukin-1β and TNF-a (66–69). However, it has been reported that IL-4 expressed in LPS-activated microglia contributes to striatal neurodegeneration, in which M1/M2 polarization is implicated, and the neutralizing antibody for IL-4 can protect striatal neurons against LPS-induced neurotoxicity *in vivo* (70). Also, we noticed increased IL-10 expression in the striatum of individual TS, despite its well-known anti-inflammatory factor. A possible reason is that IL-10 can be produced by other neural cells (e.g., Treg cells) to modulate microglia to the M2 phenotype (71, 72). However, the above speculations lack direct evidence.

In conclusion, microglia activation is M1 hyperpolarized, which transmits inflammatory injury to striatal dopaminergic neurons and disrupts normal dopamine signaling in the striatum of TS rats.

## Data availability statement

The raw data supporting the conclusions of this article will be made available by the authors, without undue reservation.

## Ethics statement

The animal study was reviewed and approved by The ethics committee of Fujian Maternity and Child Health Hospital, Affiliated Hospital of Fujian Medical University.

## Author contributions

XW and XL made substantial contributions to the design of the present study. XW, LC, and XZ collected and investigated the data. The data analysis was performed by XW and XL. XW, LC, and XZ drafted the work. XL critically revised the manuscript for important intellectual content. All authors contributed to the article and approved the submitted version.
